# Non-Classical monocytes display inflammatory features: Validation in Sepsis and Systemic Lupus Erythematous

**DOI:** 10.1038/srep13886

**Published:** 2015-09-11

**Authors:** Ratnadeep Mukherjee, Pijus Kanti Barman, Pravat Kumar Thatoi, Rina Tripathy, Bidyut Kumar Das, Balachandran Ravindran

**Affiliations:** 1 Infectious Disease Biology Group, Institute of Life Sciences, Bhubaneswar, India.; 2 Department of Medicine, S. C. B. Medical College, Cuttack, India.; 3 Post Graduate Department of Pediatrics, Sishu Bhawan, Cuttack, India

## Abstract

Given the importance of monocytes in pathogenesis of infectious and other inflammatory disorders, delineating functional and phenotypic characterization of monocyte subsets has emerged as a critical requirement. Although human monocytes have been subdivided into three different populations based on surface expression of CD14 and CD16, published reports suffer from contradictions with respect to subset phenotypes and function. This has been attributed to discrepancies in reliable gating strategies for flow cytometric characterization and purification protocols contributing to significant changes in receptor expression. By using a combination of multicolour flow cytometry and a high-dimensional automated clustering algorithm to confirm robustness of gating strategy and analysis of ex-vivo activation of whole blood with LPS we demonstrate the following: a. ‘Classical’ monocytes are phagocytic with no inflammatory attributes, b. ‘Non-classical’ subtype display ‘inflammatory’ characteristics on activation and display properties for antigen presentation and c. ‘Intermediate’ subtype that constitutes a very small percentage in circulation (under physiological conditions) appear to be transitional monocytes that display both phagocytic and inflammatory function. Analysis of blood from patients with Sepsis, a pathogen driven acute inflammatory disease and Systemic Lupus Erythmatosus (SLE), a chronic inflammatory disorder validated the broad conclusions drawn in the study.

Monocytes are a group of immune cells that originate in bone marrow and are released into peripheral blood, where they circulate for several days^1^,^2^. They belong to the mononuclear-phagocyte system, which also include macrophages, dendritic cells, and their bone-marrow precursors^3^,^4^,^5^. Monocytes represent 5–10% of peripheral leucocytes and are probably best known for serving as a systemic reservoir of myeloid precursors that are needed for the renewal of tissue macrophages and dendritic cells^6^,^7^,^8^,^9^. However, they also have other well documented functions in immune response against infection^10^,^11^,^12^,^13^, and in pathogenesis of several inflammatory disorders. Although initially perceived as a homogeneous population, it has become increasingly apparent that monocytes display considerable heterogeneity with respect to their phenotype and function^1^,^2^,^14^,^15^.

In humans, monocytes have been divided into three subtypes based on relative surface expression of LPS co-receptor CD14 and FCγIII receptor CD16^16^,^17^. The most predominant of the three, termed “classical monocytes”, express high levels of CD14 on their surface, are devoid of surface CD16, and account for approximately 80% of the total monocyte population. The remaining 20% express CD16 and have been further classified into two subtypes. The more abundant “nonclassical monocytes”, are characterized by very low expression of surface CD14 and high levels of CD16, whereas the third monocyte subtype, called “intermediate monocytes”, express high levels of both the receptors^16^,^17^.

Over the years, a number of studies, often contradictory, aimed at functional characterization of the three monocyte subsets have been undertaken by different research groups^18^,^19^,^20^,^21^,^22^,^23^,^24^,^25^,^26^, which has sparked an interest to investigate proportions of monocyte subsets in a wide variety of diseases. Relative percentages of monocyte subsets has been reported to understand pathogenesis of several infectious and metabolic diseases viz., sepsis^27^,^28^,^29^, chronic liver disease^30^,^31^,^32^, rheumatoid arthritis^33^, atherosclerosis^34^, filariasis^35^ and obesity^36^. However, lack of consistent gating strategies for quantification by flow cytometry has been a major limitation, leading to lack of reproducibility. Since CD16 is highly expressed on neutrophils and NK cells, these cell types need to be excluded from analysis for reproducible quantification and to avoid confounders. The other major limiting factor is that all functional studies so far have been performed with purified monocytes, contributing to highly variable results depending on method of isolation employed^37^.

In this study, we used a combination of multicolour flow cytometry, multiparameter imaging cytometry and a high-dimensional automated clustering algorithm to confirm robustness of our gating strategy. Further, we have addressed these controversies on analysis of functional phenotype of human monocyte subsets in circulation. We demonstrate that gradient purified monocytes are significantly different from cells analysed in whole blood for receptor expression. Our findings suggest that ficoll purification of blood monocytes leads to a decrease in number of CD14+/CD16- classical monocytes with a concomitant expansion of CD14dim/CD16+ nonclassical monocytes. Moreover, gradient separation also contributed to increased surface expression of CD16. Therefore, in order to retain physiologically relevant monocyte function, we adopted whole blood stimulation and staining protocol. Using this method we show distinct phenotype and function of monocyte subsets in circulation with respect to inflammation, antigen presentation and phagocytosis. We unequivocally demonstrate that nonclassical and not intermediate subtype are the primary inflammatory monocytes and further validate by studying patients suffering from sepsis and systemic lupus erythematous (SLE).

## Results

### Identification of monocyte subsets in human blood

**Figure 1:**
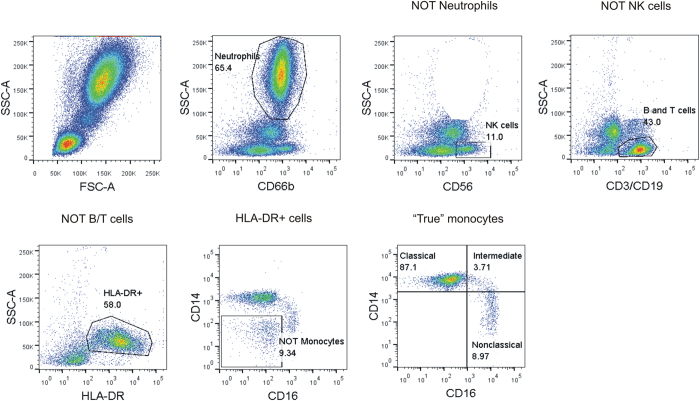
Identification of blood monocyte subsets by polychromatic flow cytometry. Gating strategy for identification of monocyte subsets showing successive exclusion of neutrophils, NK cells, B and T cells on conventional bivariate scatterplots of side scatter signal vs. exclusion marker specific for cell type. Remaining population was further selected for HLA-DR which was discriminated on a CD14 vs. CD16 scatterplot to give three monocyte subsets.

To properly identify monocyte subpopulations in blood, we employed a negative “exclusion” gating strategy. Apart from monocytes, neutrophils and NK cells also express high levels of CD16. Therefore, to avoid scoring of false positive events, we excluded these two cell types by using Boolean NOT gates on a bivariate plot of side scatter signal vs. cell surface receptor expression specific for neutrophils and NK cells (Fig. 1). CD3 positive T cells and CD19 positive B cells were similarly excluded. Leftover cells were further selected for ‘true’ monocytes by gating on HLA-DR positive events. The resulting final population was discriminated on CD14 and CD16 surface expression to give three distinct monocyte subsets, about 90% of which was the ‘classical’ CD14+/CD16- subtype. ‘Intermediate’ CD14+/CD16+monocytes constituted 2–3% of total monocytes, while about 7–8% was made up by the ‘nonclassical’ CD14dim/CD16+subtype (Fig. 1).

**Figure 2:**
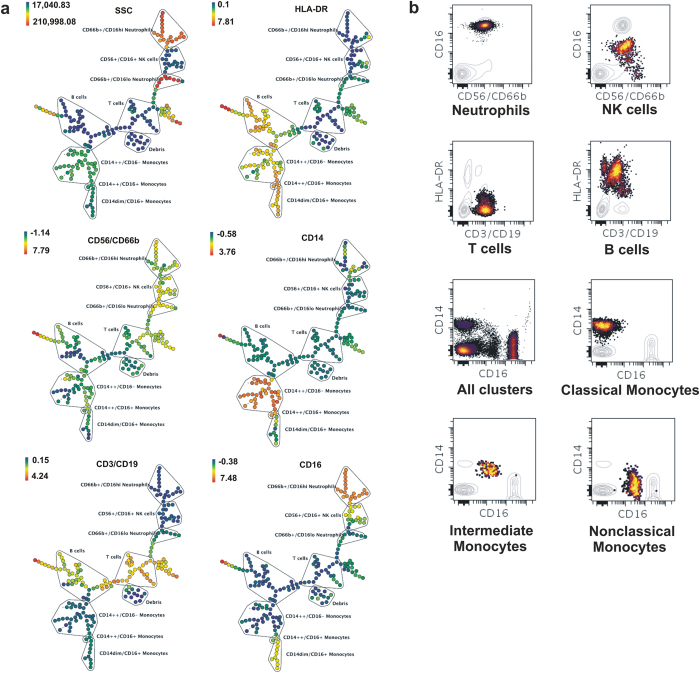
SPADE analysis of circulating immune cells showing validity of manual gating. Confirmation of manual gating strategy by SPADE implemented as a web-based extension of Cytobank. Number of nodes was set at 200 with 10 per cent downsampling of events. (**a**) Each “bubble” on a given tree corresponds to a particular cell-type that was selected according to expression of its lineage marker as indicated by the colour code (expression decreasing from red to blue). (**b**) Associated bivariate scatterplots confirm the gating strategy by highlighting only a selected bubble at a time while greying out other populations.

Manual gating in flow cytometry is often subjective that can lead to considerable variation in identification of rare cell types at the hands of different users^38^. Keeping this in view, we used an automated clustering algorithm called SPADE (Spanning-Tree Progression Analysis of Density-normalised Events) to confirm our gating strategy. SPADE is a versatile computational tool for visualization of high-dimensional flow cytometry data^38^. It involves density-dependent downsampling of raw flow cytometry data followed by agglomerative clustering based on relative expressions of different cellular antigens. This leads to construction of minimum spanning trees connecting the clusters that allows easy visualization of rare events. We implemented SPADE as a web-based extension of Cytobank with default settings of 200 nodes and 10 per cent downsampling of events. The resulting clustering trees are shown in Fig. 2a. SPADE analysis was able to clearly distinguish circulating immune cells based on surface antigen expression denoted by a colour gradient (Fig. 2a). The efficiency of SPADE is further demonstrated by a comparison of the clusters with their counterparts on conventional bivariate scatterplots (Fig. 2b) that show similarities with manually gated populations depicted in Fig. 1.

**Figure 3:**
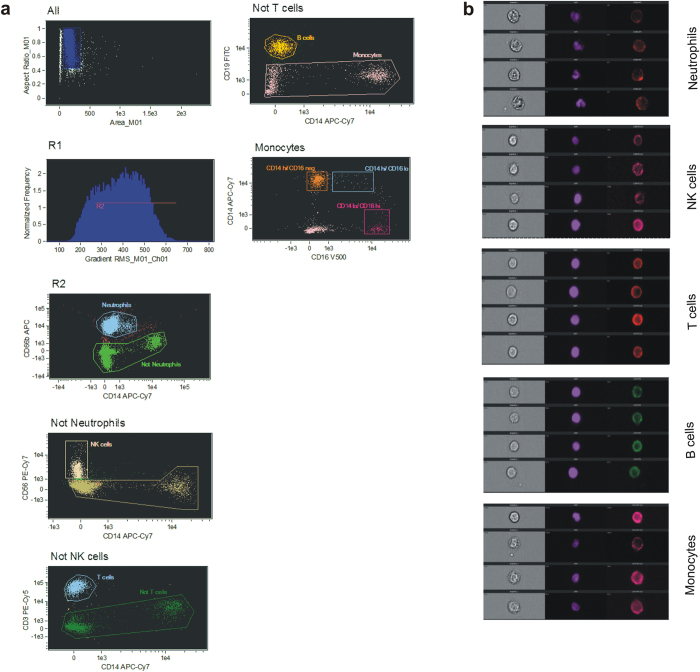
Multispectral imaging cytometry based visualization and identification of circulating immune cells. (**a**) Single cells were selected first (R1) followed by gating of cells in focus (R2). Thereafter, stepwise negative selection of nonmonocyte populations was done to finally include “true” monocytes. (**b**) Representative brightfield (left), nuclear (middle) and surface antigen (right) images of each different cell type as gated in (**a**) confirming individual cell types based on nuclear morphology, brightfield image and lineage marker expression.

As a third approach, we utilised flow cytometry based high resolution imaging to further confirm our gating strategy. This technology allows visualization of cellular morphology after gating individual cell types using conventional bivariate scatter plots. As shown in Fig. 3a, acquired events were first gated based on area and aspect ratio of brightfield image to select single cells, followed by which blurred images were “gated out” in the next plot. The resulting events were analysed according to the exclusion gating strategy described previously. It is clear from Fig. 3b that immune cell types in circulation can be easily differentiated using a combination of bright field, nuclear and signature antigen specific images. This further validated our gating strategy by visibly demonstrating that contribution of contaminating cell populations in our analyses were negligible.

### Density gradient centrifugation purification of PBMC changes surface expression of CD14 and CD16 on monocytes

**Figure 4:**
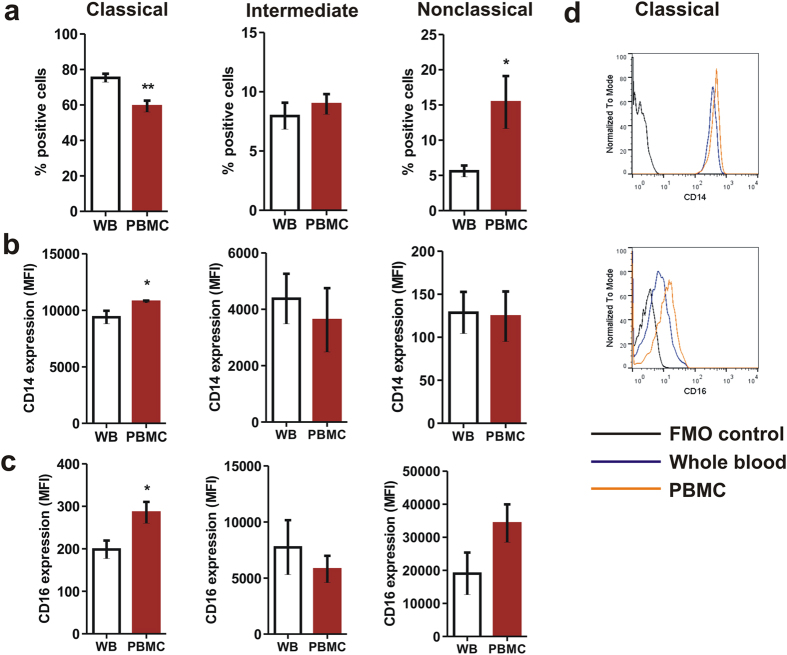
Gradient purification of PBMCs from whole blood leads to changes in monocyte phenotype. Comparison of monocyte subsets between whole blood monocytes and ficoll purified monocytes showing (**a**) significant decrease in proportion of ‘classical’ monocytes and increase in ‘nonclassical’ monocytes as a consequence of gradient purification that also led to increase in (**b**) CD14 and (**c**) CD16 expression on classical monocytes. Statistical significance was ascertained using a paired t-test. (**d**) Representative overlaid histograms showing increased expression of CD14 and CD16 on classical monocyte surface as a consequence of Ficoll purification. Data are mean ± SEM of five normal subjects. *P < 0.05, **P < 0.01. MFI: Median Fluorescence Intensity.

One of the major issues with earlier studies involving analysis of monocyte subset phenotype and function has been use of *in vitro* purified monocytes. It has been suggested by different investigators that inconsistent results could have been due to purification protocols followed^39^,^40^. Therefore, we compared flow cytometry analysis of monocytes from purified PBMC and whole blood. Using the gating strategy mentioned above profound changes was observed in both subset percentage and surface expression of CD14 and CD16. Purification in PBMCs led to a significant decrease in CD14+/CD16- monocytes with a concurrent increase in CD14dim/CD16+monocytes (Fig. 4a). Purification of PBMC also significantly upregulated surface expression of CD16 on CD14+/CD16- monocytes (Fig. 4c, *left panel*).

Collectively, above findings indicate that purification of PBMC from whole blood leads to variations in surface expression of CD14 and CD16 which may lead to incorrect observations involving such purified subsets. Therefore, we used whole blood stimulation and analysis of monocyte subtypes in order to infer physiologically meaningful conclusions.

### Monocyte subsets have distinctive patterns of cell surface receptor expression

**Figure 5:**
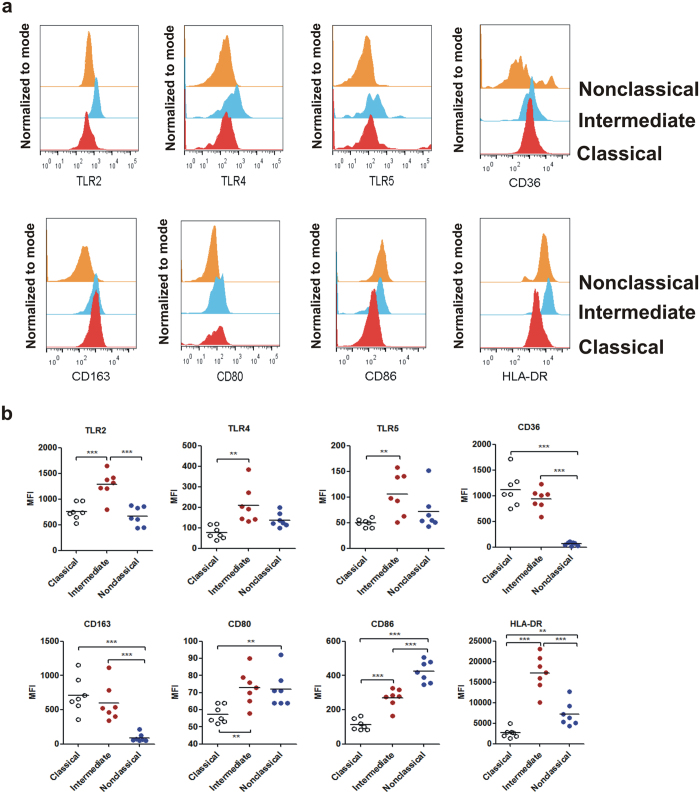
Differential expression of surface receptors on monocyte subsets. (**a**) Representative overlaid histograms of eight different surface markers showing differential expression between monocyte subsets. Histograms were created using FlowJo software. (**b**) Different monocyte subtypes showing expression of a specific repertoire of surface antigens. Assessment of statistical significance was performed by one-way ANOVA followed by Bonferroni’s post-test. Data are mean ± S.E.M. of seven normal subjects. *P < 0.05, **P < 0.01, ***P < 0.001. MFI: Median Fluorescence Intensity.

Having established a proper gating strategy to identify monocyte subsets in humans as well as showing that physiologically relevant inferences pertaining to their phenotype and function can be drawn only by studying them *ex vivo* with as little manipulation as possible, we wanted to check surface expression patterns of toll-like receptors, phagocytic receptors and co-stimulatory molecules on monocyte subsets. Our data show that CD14+/CD16+“intermediate” monocytes express significantly higher levels of TLRs 2, 4 and 5 as compared to the other two subsets, indicating a primarily proinflammatory function (Fig. 5a,b, *top panel*). This subtype also expresses high levels of CD80, CD86 and HLA-DR that is suggestive of a role in antigen presentation (Fig. 5a,b, *bottom panel*). However, the “nonclassical” CD14dim/CD16+ subset were also found to express significantly high levels of CD80 and CD86, indicating high antigen presenting capability (Fig. 5a,b, *bottom panel*). Interestingly, the CD14+/CD16- “classical” monocytes expressed significantly low levels of TLRs and co-stimulatory molecules while expressing highest levels of CD36 and CD163, thereby suggesting that majority of blood monocytes are primarily phagocytic in nature (Fig. 5a,b, *top and bottom panel*).

Our next aim was to check for changes in expression patterns of surface receptors on monocytes as a result of activation. To this end, we stimulated whole blood with gram negative bacterial LPS for 4 hours and scored surface receptor expression by flow cytometry. Stimulation of whole blood with LPS did not significantly alter the proportion of the three monocyte subsets (Supplementary Fig. 1a) other than upregulating receptors on all of them (Supplementary Fig. 1b).

Taken together, above data indicate that human blood monocyte subsets have different repertoire of surface receptors that may dictate specific functional roles.

### Assessment of blood monocyte subset function

**Figure 6:**
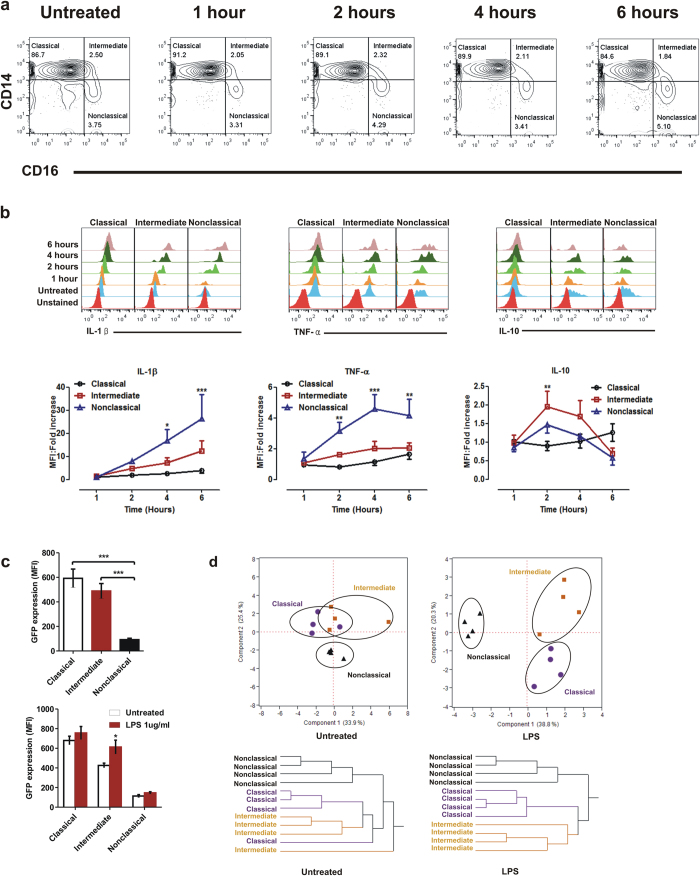
Functional analysis of human monocyte subsets. (**a**) Representative contour plots of monocyte subsets showing unchanged relative percentages even after 6 hours of LPS stimulation. (**b**) Analysis of cytokine production by monocyte subsets. Whole blood was left untreated or treated with LPS for the indicated time points along with Brefeldin A at a final concentration of 3 μg/ml to prevent secretion and stained for intracellular cytokines along with surface markers for exclusion gating as described previously. Brefeldin A was added one hour prior to termination of culture at each time point. Top panel shows representative overlaid histograms whereas bottom panel data are represented as mean ± SEM of normalized fold change over untreated samples obtained from five donors. *P < 0.05, **P < 0.01, ***P < 0.001 from one-way ANOVA followed by Bonferroni’s post-test. MFI: Median Fluorescence Intensity. (**c**) Differential phagocytic function of monocyte subsets as demonstrated by uptake of GFP-expressing *E.coli* under normal conditions as well as post activation with LPS. Whole blood was incubated with or without live bacteria for 30 minutes followed by staining for surface markers. Statistical significance was assessed either by one-way (*left panel*) or two-way (*right panel*) ANOVA followed by Bonferroni’s post-test. *P < 0.05, **P < 0.01, ***P < 0.001. (**d**) Principal components analysis and hierarchical clustering of untreated and LPS stimulated monocytes showing relationship between three monocyte subsets. Median fluorescence intensities obtained from CD14, CD16, TLR2, TLR4, TLR5, CD80, CD86, CD36, CD163, HLA-DR, IL-1β, TNF-α and IL-10 staining were used as parameters for principal components analysis and clustering.

Nature of cytokines produced by different monocyte subsets has been a major source of controversy in literature. We, therefore, sought to resolve this contentious issue by stimulating whole blood with LPS and quantified intracellular cytokines. ‘Non-classical’ monocytes were the primary producers of inflammatory cytokines IL-1β and TNF-α (Fig. 6b) while intermediate monocytes produced mostly IL-10 (Fig. 6b). These findings are in stark contrast to published reports that classify ‘intermediate’ monocytes as inflammatory and ‘classical’ monocytes as the primary producers of IL-10. We attribute this discordance to experimental artefacts associated with *in vitro* purified monocytes, a possibility suggested by other investigators. Interestingly, ‘intermediate’ monocytes showed significantly higher levels of intracellular IL-1β and TNF-α at steady-state compared to ‘non-classical’ monocytes (Supplementary Fig. 2), an observation that is in concordance with higher toll-like receptor expression on ‘intermediate’ monocytes (Fig. 5). It is worth mentioning here that several investigators have reported a loss of CD16 expression upon stimulation of whole blood with LPS leading to problems with identification of the non-classical subset^26^,^41^**. In our study, however, we did not find any loss of CD16 expression even after 6 hours of LPS stimulation at which the non-classical subset was clearly identifiable (**Fig. 6a).

*In situ* phagocytic function using GFP-E. coli revealed ‘Classical’ subset and to some extent ‘intermediate’ monocytes to be highly phagocytic (Fig. 6c)—an outcome predictable based on increased expression of scavenger receptors on these subsets (Fig. 5). This observation in accordance with published reports suggests that primary function of majority of blood monocytes is phagocytosis.

To predict a relationship between monocyte subsets, we pooled all expression data obtained thus far and subjected them to principal components analysis and hierarchical clustering (Fig. 6d). Our model illustrates a close relationship between classical and intermediate subsets, with the nonclassical subset forming a distant cluster.

### Non-classical monocyte subset percentage increases during inflammatory diseases

**Figure 7:**
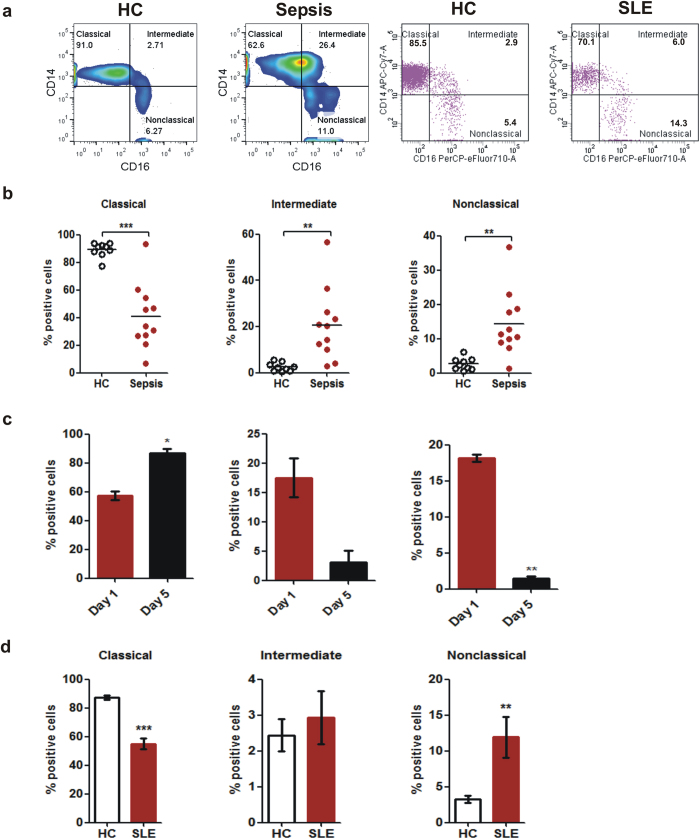
Expansion of CD16 positive monocytes during inflammatory diseases. (**a**) Representative bivariate scatterplots of monocyte subset percentage showing comparison of sepsis and SLE patients with their respective age and sex matched healthy controls. (**b**) Comparison of monocyte subsets (percentage) between healthy subjects (n = 9) and Sepsis patients (n = 11). (**b**) Analysis of monocyte subsets on Day 1 and Day 5 (post treatment) of Sepsis patients (n = 3). (**c**) Comparison of monocyte subsets (percentage) between healthy subjects (n = 13) and SLE patients (n = 10). *P < 0.05, **P < 0.01, ***P < 0.001 assessed by unpaired t-test.

To validate our conclusion that non-classical monocytes are indeed the dominant ‘inflammatory’ subtype we analysed monocyte subpopulations in two groups of patients with distinct display of systemic inflammation viz., Sepsis and SLE. Sepsis is associated with pathogenic infection while SLE is a chronic autoimmune disease of unknown aetiology. A significant increase of both ‘intermediate’ and ‘non-classical’ subsets was observed in Sepsis patients while only ‘non-classical’ monocytes were increased in SLE patients when compared with normal controls (Fig. 7a,c). Following treatment of Sepsis patients ‘non-classical’ inflammatory monocytes recovered to normal levels (Fig. 7b). Expression of TLRs, SRs and co-stimulatory molecules was significantly upregulated in all monocyte subsets in Sepsis patients (Supplementary Fig. 3), a feature observed on *ex vivo* LPS stimulation of normal human blood (Supplementary Fig. 1b). However, there was no significant relationship between plasma levels of TNF-α and IL-10 in representative samples of sepsis cases (Supplementary Fig. 4), a finding not unexpected as plasma cytokine levels are a sum of many different contributing cell types and cannot be attributed to a specific cell type alone. Moreover, stability of different cytokines in plasma is also variable, thus making association of plasma cytokines to intracellular cytokines difficult.

## Discussion

Existence of heterogeneity in human peripheral blood monocytes based on size and density was reported almost three decades ago, showing that human monocytes in circulation consisted principally of two subtypes – a large and a small sized one – that differed in their phagocytic and inflammatory capabilities^42^,^43^,^44^,^45^,^46^,^47^. Later, with the advent of flow cytometry, a more robust identification of blood monocyte subsets was performed based on differential expression of surface CD14 and CD16^48^. This initial study showed existence of at least two distinct monocyte subsets – CD14+/CD16- and CD14+/CD16+- that were strikingly different in their function. Subsequently, the CD16+monocytes were shown to be further composed of two different populations^14^. One was shown to express equal levels of CD14 and CD16 (CD14+/CD16+), while the other population was characterized by very low surface expression of CD14 (CD14dim/CD16+).Given the importance of monocytes in immune function and their role in pathogenesis of a wide variety of diseases, it became essential to precisely characterize human blood monocyte subsets. To this end, a large number of studies were undertaken by different research groups with a view to functionally characterize blood monocyte subsets^18^,^19^,^20^,^21^,^22^,^23^,^24^,^25^,^49^. However, there exists little consensus among most of published literature. One of the reasons is lack of a consistent gating strategy for immunophenotyping by flow cytometry. This issue was addressed by a recent review that proposed a common gating strategy to identify monocyte subpopulations in human circulation^16^. Accordingly, in the current study, a negative gating strategy was used to sequentially exclude neutrophils, NK cells, B cells and T cells from analysis followed by inclusion of HLA-DR+++monocytes that were further discriminated on a bivariate scatterplot of CD14 vs. CD16 to finally yield three clearly distinguishable monocyte subpopulations. According to proposed nomenclature^16^,^17^, they were classified as ‘classical’ (CD14+/CD16-), ‘intermediate’ (CD14+/CD16+), and ‘nonclassical’ (CD14dim/CD16+). In accordance with previously published reports, classical monocytes were the most numerous, constituting about 80–90% of blood monocytes, with intermediate and nonclassical subtypes making up the rest.

A contentious issue with analysis of high dimensional flow cytometry data is the use of manual gating which is subjective and relies on visual inspection that varies between users leading to errors^50^,^51^. This problem is especially evident during identification of rare cell types. Therefore, to validate our gating strategy we employed an automated clustering algorithm called spanning tree progression analysis of density-normalized events (SPADE)^38^. SPADE involves density-dependent downsampling of raw flow cytometry data followed by agglomerative clustering based on relative expressions of different cellular antigens. This leads to construction of minimum spanning trees connecting the clusters that allows easy visualization of rare events. The similarities between bivariate plots created through SPADE and manual gating validated our manual gating strategy. A further confirmation of robustness of manual gating was obtained through high resolution imaging cytometry in which each individual population of cell was visualized and were clearly distinguishable via a combination of cell surface marker expression and nuclear morphology.

A growing body of opinion suggests that the primary reason for discrepancies between previously published reports on monocyte subset function is use of *in vitro* purified monocytes in culture and that whole blood stimulation and analysis could be a better alternative^39^,^40^. A number of studies have shown benefits of whole blood culture and stimulation over purified cells for *in vitro* analysis of immune cell function^37^,^52^,^53^,^54^,^55^. In the present study, we observed that purification of peripheral blood mononuclear cells (PBMCs) by ficoll-density gradient centrifugation led to considerable changes in relative percentages of monocyte subtypes - classical monocytes decreased with a simultaneous increase in percentage of nonclassical monocytes. When compared with whole blood analysis, our results provide evidence to the notion that gradient purification of monocytes can lead to experimental artefacts that can confound analysis of monocyte function.

Analysis of surface molecules on monocyte subpopulations revealed differential expression of toll-like receptors (TLRs), scavenger receptors and co-stimulatory molecules among the three monocyte subsets. Intermediate and nonclassical monocyte subsets expressed more TLRs 2, 4, 5, co-stimulatory molecules CD80, CD86 and HLA-DR than the classical subset, suggesting their role in antigen presentation. On the other hand, higher expression of scavenger receptors CD36 and CD163 on classical monocytes was suggestive of their predominantly phagocytic function. Our observation that CD80 and CD86 is differentially expressed among monocyte subsets is in disagreement with an earlier published report^23^ that showed no difference in expression of CD80 and CD86 in purified monocyte subsets. Interestingly, the observed difference in expression of these two molecules within monocyte subsets was no longer present following stimulation of monocytes with LPS, suggesting that the earlier observation may have been a consequence of activation during purification process.

A constant source of controversy regarding function of subsets of human monocyte the type of cytokines produced by them. While inflammation is a complex phenomenon involving several receptors, mediators and pathways, monocytes that synthesize molecules such as TNF-α, IL-1β, IL-6 etc. have been classified and designated as ‘inflammatory subtype’ by us in this manuscript. The current study demonstrates that nonclassical monocytes are the primary producers of TNF-α and IL-1β upon activation as shown by intracellular cytokine staining. This observation is markedly different from an earlier observation^21^ that classified intermediate monocytes to be primarily responsible for inflammatory cytokine production while the non-classical monocytes showed patrolling behaviour. It is pertinent to note that these authors had used bead purified monocytes *in vitro* and measured released cytokines in supernatants. Another interesting observation was increased IL-10 production by intermediate subsets, which differs from earlier studies that demonstrated classical monocytes as principal producers of IL-10^21^,^23^. While whole blood assays performed in this study is closer to physiological condition (unlike bead purified monocyte subsets) the discrepancy between earlier studies^21^,^23^ and our current observation could also be due to difference in the assay system adapted for measuring cytokines. While the previous reports had measured secreted cytokines by ELISA in the current study we have quantified intracellular cytokines by flow cytometry. Intracellular cytokines do not necessarily correlate with secreted cytokines, particularly in the case of IL-1β secretion which is tightly regulated. Thus the observed differences in TNF-α, IL-1β and IL-10 positive monocyte subsets could be a result of the two different assay systems used in the investigations. Another probable cause could be a result of observations made at different time points as most studies reported in literature were all 18 hour stimulations followed by analysis of cytokines whereas our observations were at much earlier times points.

There was concordance between scavenger receptor expression and functional phagocytosis in different monocyte subsets. Classical and intermediate monocytes were found to be highly phagocytic while nonclassical monocytes were poorly phagocytic, an observation in agreement with previous reports^21^. Our data also provide circumstantial evidence to the hypothesis that intermediate monocytes may not be a distinct endpoint of differentiation rather a developmental stage between classical and nonclassical subsets. Principal components analysis and hierarchical clustering revealed the intermediate and classical monocytes to be closely linked whereas the nonclassical subset formed a distant cluster. This close relationship between intermediate and classical subsets has been suggested earlier^21^. It would be of interest to investigate if intermediate monocytes can switch to-and-fro between classical and nonclassical subtypes, i.e. between a predominantly phagocytic and a primarily inflammatory phenotype depending on different activation cues.

Flow cytometry-based detection of immune cell types in the context of various diseases is a useful way to diagnose severity and outcome in a clinical setting. We validated our findings in two inflammatory disease conditions, viz. sepsis and SLE. Our results indicate that expansion of CD16+ monocytes can be used to determine an inflammatory condition that is consistent with published reports^27^,^28^,^29^,^33^. However, a question that remained unanswered was which one of the CD16+ monocyte subsets expand during inflammation? Our data show that while in an acute inflammation like sepsis both subsets increase in percentage with a concomitant decrease in classical monocyte percentage, in a chronic inflammatory situation like SLE, only the nonclassical subset is expanded. Although it is not clear whether such increase in inflammatory cell types is a cause or consequence of disease, it tends to suggest that increase in intermediate monocytes could be a differentiating factor between acute and chronic inflammation.

We conclude that ‘Classical’ monocytes are phagocytic with no inflammatory attributes, ‘Non-classical’ subtype display ‘inflammatory’ characteristics on activation and exhibit properties for antigen presentation while ‘Intermediate’ monocytes constitute a very small percentage in circulation (under physiological conditions) and appear to be a minor transitional subset that displays both phagocytic and inflammatory function. Given the importance of understanding monocyte subtypes in several human diseases (infectious as well as metabolic) very large number of reports have begun to clutter medical literature using un-validated flow cytometric assays. This highly validated phenotypic and functional characterization of Human monocyte subtypes should bring clarity to investigators in human immunology.

## Materials and Methods

### *Ex vivo* stimulation of human peripheral blood

Whole blood was withdrawn from apparently healthy donors by venepuncture in acid citrate dextrose (ACD) anticoagulant at 15% v/v. Donors were recruited from institute students. For scoring expression of surface markers, fifty microlitre blood was incubated with or without gram-negative bacterial LPS (*E. coli* O55:B5, Sigma Aldrich) at 1 μg/ml for 4 hours in a 37 °C water bath. To test for differential cytokine expression between monocyte subsets, fifty microlitre whole blood was left untreated or stimulated with LPS at 1 μg/ml for 1, 2, 4 or 6 hours in 37 °C water bath along with brefeldin A (eBiosciences) at 1:1000 dilution. Post stimulation, cells were stained with fluorochrome conjugated antibodies and analysed on a flow cytometer.

### Patient recruitment

The study was approved by ethics committees of Institute of Life Sciences and S.C.B. Medical College, and signed informed consent was obtained from all participants. We included 11 sepsis patients who were admitted to medical intensive care units and 10 SLE patients admitted at the Department of Medicine, S.C.B. Medical College and Hospital (Cuttack, India). Table 1 summarizes the demographic and clinical characteristics of the patients. For sepsis, patients were eligible for inclusion only if they had systemic inflammatory response syndrome and had a source of infection, proven or suspected. We used the acute physiological and chronic health evaluation II (APACHE II) scoring system to categorize the patients. The definitions of sepsis, severe sepsis, septic shock, and multi-organ dysfunction syndrome (MODS) were in accordance with published criteria^56^,^57^. The following categories of patients were excluded from the study: patients with diabetes mellitus, hypertension, nephrotic syndrome, chronic kidney disease (sonographic feature of CKD and/or GFR < 30 ml/min), patients with cardiac failure and immunocompromised individuals.

### Staining for cell surface markers

Stimulated whole blood was incubated for 30 min at 4 °C with fluorescently tagged monoclonal antibodies (Supplementary Table I). One millilitre of 1X FACS Lysing solution (BD Biosciences) was added and incubated at room temperature for 20 min to lyse red blood cells. Following RBC lysis, cells were washed twice with two millilitre ice-cold PBS at 400X g for 5 min. Cells were resuspended in five hundred microliter PBS and acquired on a BD LSR Fortessa flow cytometer (BD Biosciences).

### Intracellular cytokine staining

For staining of surface antigens, appropriate antibody cocktails were added 1 hour before termination of stimulation and incubated at 37 °C. One millilitre of 1X Lyse/Fix buffer (BD Biosciences) was added to each tube with gentle vortexing and incubated at 37 °C water bath for 10 min. After this fixation/lysis step, blood was washed twice with two milliletre cold PBS followed by incubation with five hundred microlitre 1X permeabilization buffer IV (BD Biosciences) for 20 min at room temperature. Post permeabilization, fluorochrome conjugated antibodies specific to intracellular cytokines (Supplementary Table I) were added and samples incubated at room temperature in the dark for 30 min. The cells were washed with two millilitre cold PBS, resuspended in five hundred microliter PBS and acquired on a BD LSRFortessa flow cytometer.

### Phagocytosis assay

GFP-expressing *E.coli* was added to whole blood left untreated or treated with LPS for 2 hours at a density of 50 bacteria per cell. After incubation at 37 °C for 30 min, samples were stained with a cocktail of cell surface antibodies, RBCs were lysed, washed and analysed on a flow cytometer.

### Flow cytometry

All samples were analysed on an LSRFortessa flow cytometer (Becton Dickinson) fitted with 355 nm, 488 nm and 640 nm laser lines. PMT gain for all fluorescence channels were set with FACS Diva software (v7.0) using robust standard deviation of electronic noise (rSDEN) for each detector obtained from BD Cytometer Setup and Tracking (CS&T) beads. Spectral spillover of fluorophore signals into detectors other than its own was corrected by creating an autocompensation matrix generated by singly stained compensation beads (FacsComp beads, BD Biosciences). All cytometer settings were saved as experiment specific application settings which were updated by CS&T beads before each new acquisition.

10000 monocyte events were recorded for each sample based on an arbitrary gate created on a bivariate scatterplot of forward vs. side scattered light signals. For defining gates during analysis, fluorescence minus one (FMO) control tubes were used that lacked one fluorescently tagged antibody in the cocktail of all antibodies used for staining. Flow cytometry data was either analysed on FACS Diva v7.0 (BD Biosciences) or exported as FCS3.1 files and analysed in FlowJo software (TreeStar).

### Multispectral imaging cytometry

Whole human blood was stained, lysed and washed as described in previous sections. Cells were counterstained with DAPI (Molecular Probes) at a final concentration of 1.4 μM, washed and analysed on a ImagestreamX imaging cytometer (Amnis corp.) equipped with 405 nm, 488 nm, 561 nm, 594 nm and 640 nm lasers. 25,000 total events were acquired using INSPIRE acquisition software. Raw image files (.rif) were analysed with IDEAS version 4.0.

### SPADE analysis

SPADE stands for spanning tree progression analysis of density-normalised events. It is an automated clustering algorithm that allows visualization of rare events in a heterogeneous population of cells without bias involved in manual gating. SPADE analysis was performed as a web-based extension of Cytobank software with default settings of 10 per cent downsampling of events with 200 nodes.

### Statistical analysis

One-way or two-way analysis of variance was used with Bonferroni’s post hoc test to assess significance between groups. A p-value of less than 0.05 was considered statistically significant. For assessing relationships between monocyte subsets, principal components analysis (PCA) using restricted maximum likelihood (REML) estimates and hierarchical clustering by Ward’s method was employed.

## Additional Information

**How to cite this article**: Mukherjee, R. *et al.* Non-Classical monocytes display inflammatory features: Validation in Sepsis and Systemic Lupus Erythematous. *Sci. Rep.* **5**, 13886; doi: 10.1038/srep13886 (2015).

## Supplementary Material

Supplementary Information

## Figures and Tables

**Table 1:** Demographic and clinical characteristics of patients.

**Sepsis**	**Healthy Controls**	
Variable	Whole cohort (n = 11)	Whole cohort (n = 9)
Men/Women	9/2	8/1
Age, median (range), years	24 (17–75)	27 (25–32)
Pulse rate, median (range), beats/min	100 (96–130)	Normal
Respiratory rate, median (range), breaths/min	30 (16–48)	Normal
Temperature, median (range), °F	102 (100–102)	Normal
Total leucocyte count, median (range), cells/μl	14000 (7600–40000)	8000 (6000–9000)
Source of infection, n (%)		
Pneumonia	6 (54.5%)	N.A.
Urinary tract infection	5 (45.5%)	N.A.
APACHE II score, median (range)	16 (8–23)	N.A.
Mortality, n (%)	4 (37%)	N.A.
**SLE**	**Healthy Controls**	
Variable	Whole cohort (n = 10)	Whole cohort (n = 13)
Men/Women	0/10	0/13
Age, median (range), years	23 (15–42)	25 (23–33)
Fever, n (%)	9 (90%)	N.A
Arthritis, n (%)	3 (30%)	N.A
Cutaneous manifestations (%)	8(80%)	N.A
Nephritis (%)	3(30%)	N.A
Serositis (%)	5(50%)	N.A
Myocarditis (%)	1(10%)	N.A
SLEDAI score, median (range)	14 (7–27)	N.A
Glucocorticoid dose	7.5–10.0 mg/day	N.A.
