# Supplementary material for: Non-Classical monocytes display inflammatory features: Validation in Sepsis and Systemic Lupus Erythematous
Source: Sci Rep. 2015 Sep 11;5:13886. doi: 10.1038/srep13886 (PMC4566081; doi:10.1038/srep13886)
Supplement: Supplementary Information [file srep13886-s1.docx]

**Title: Non-Classical monocytes display inflammatory features: Validation in Sepsis and Systemic Lupus Erythematous**

**Authors:** Ratnadeep Mukherjee^1^, Pijus Kanti Barman^1^, Pravat Kumar Thatoi^2^, Bidyut Kumar Das^2^, Rina Tripathy^3^ & ^*^Balachandran Ravindran^1^

^1^Infectious Disease Biology Group, Institute of Life Sciences, Bhubaneswar, India.

^2^Department of Medicine, S. C. B. Medical College, Cuttack, India.

^3^Post Graduate Department of Pediatrics, Sishu Bhawan, Cuttack, India

**Correspondence:** Balachandran Ravindran, Director, Institute of Life Sciences, NALCO Square, Chandrasekharpur, Bhubaneswar–751023, Odisha, India

e-mail: [ravindran8@gmail.com](mailto:ravindran8@gmail.com)

Telephone no: 91 – 674 – 2301900

Fax:  91 – 674 – 2300728

**Supplementary Table I. Antibodies used for staining of cell surface antigens and intracellular cytokines**

| **Cell surface staining** | | | | **Intracellular staining** | | |
| --- | --- | --- | --- | --- | --- | --- |
|  | | | |  | | |
| Panel | **Antigen** | **Fluorochrome** | **Supplier** | **Antigen** | **Fluorochrome** | **Supplier** |
|  |  |  |  |  |  |  |
| Common | CD3 | Brilliant Violet 510 | BD Biosciences | CD3 | Brilliant Violet 510 | BD Biosciences |
|  | CD19 | Brilliant Violet 510 | BD Biosciences | CD19 | Brilliant Violet 510 | BD Biosciences |
|  | HLA-DR | PE-CF594 | BD Biosciences | HLA-DR | PE-CF594 | BD Biosciences |
|  | CD56 | PE-Cy7 | BD Biosciences | CD56 | Brilliant UV  395 | BD Biosciences |
|  | CD66b | PE-Cy7 | eBiosciences | CD66b | PerCP-Cy5.5 | BD Biosciences |
|  | CD16 | Alexa Fluor 700 | BD Biosciences | CD16 | Alexa Fluor 700 | BD Biosciences |
|  | CD14 | APC-H7 | BD Biosciences | CD14 | APC-H7 | BD Biosciences |
| Panel 1 | TLR2 | Alexa Fluor 488 | BD Biosciences | IL-1β | FITC | BD Biosciences |
|  | TLR5 | PE | Imgenex | IL-10 | PE | BD Biosciences |
|  | TLR4 | APC | eBiosciences | TNF-α | PE-Cy7 | Invitrogen |
| Panel 2 | CD80 | FITC | eBiosciences |  |  |  |
|  | CD163 | PE | eBiosciences |  |  |  |
|  | CD86 | PerCP-eFluor 710 | eBiosciences |  |  |  |
|  | CD36 | APC | BD Biosciences |  |  |  |

**
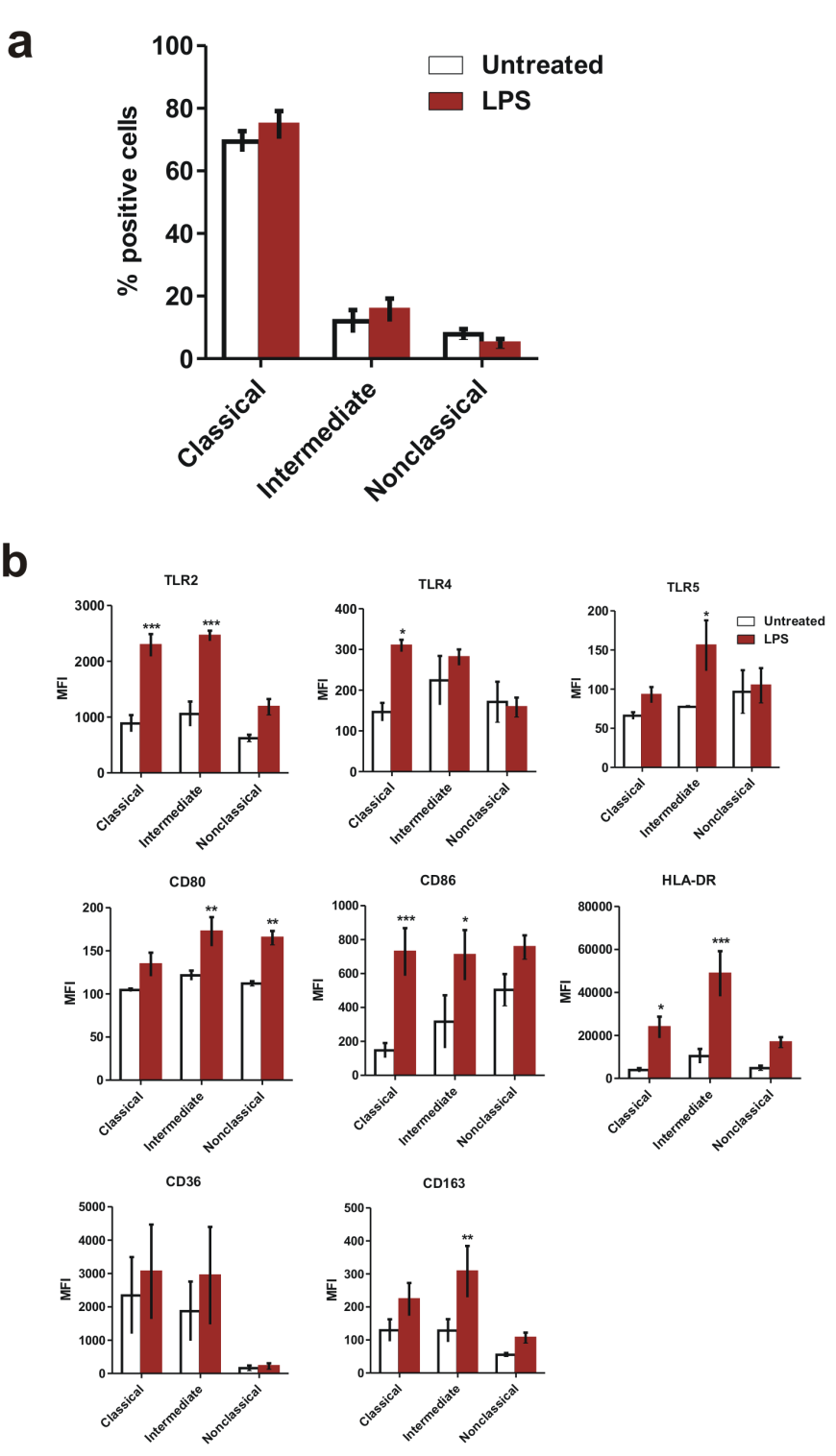
**

**Supplementary figure 1. Analysis of monocyte subset percentage and receptor expression following LPS activation.** Whole blood was left untreated or treated with LPS for 4 hours followed by staining for surface receptors and analysed for changes in monocyte subset percentages (A) and expression of surface receptors (B). **P*<0.05, ***P*<0.01, ****P*<0.001 assessed by two-way ANOVA followed by Bonferroni’s post-test.


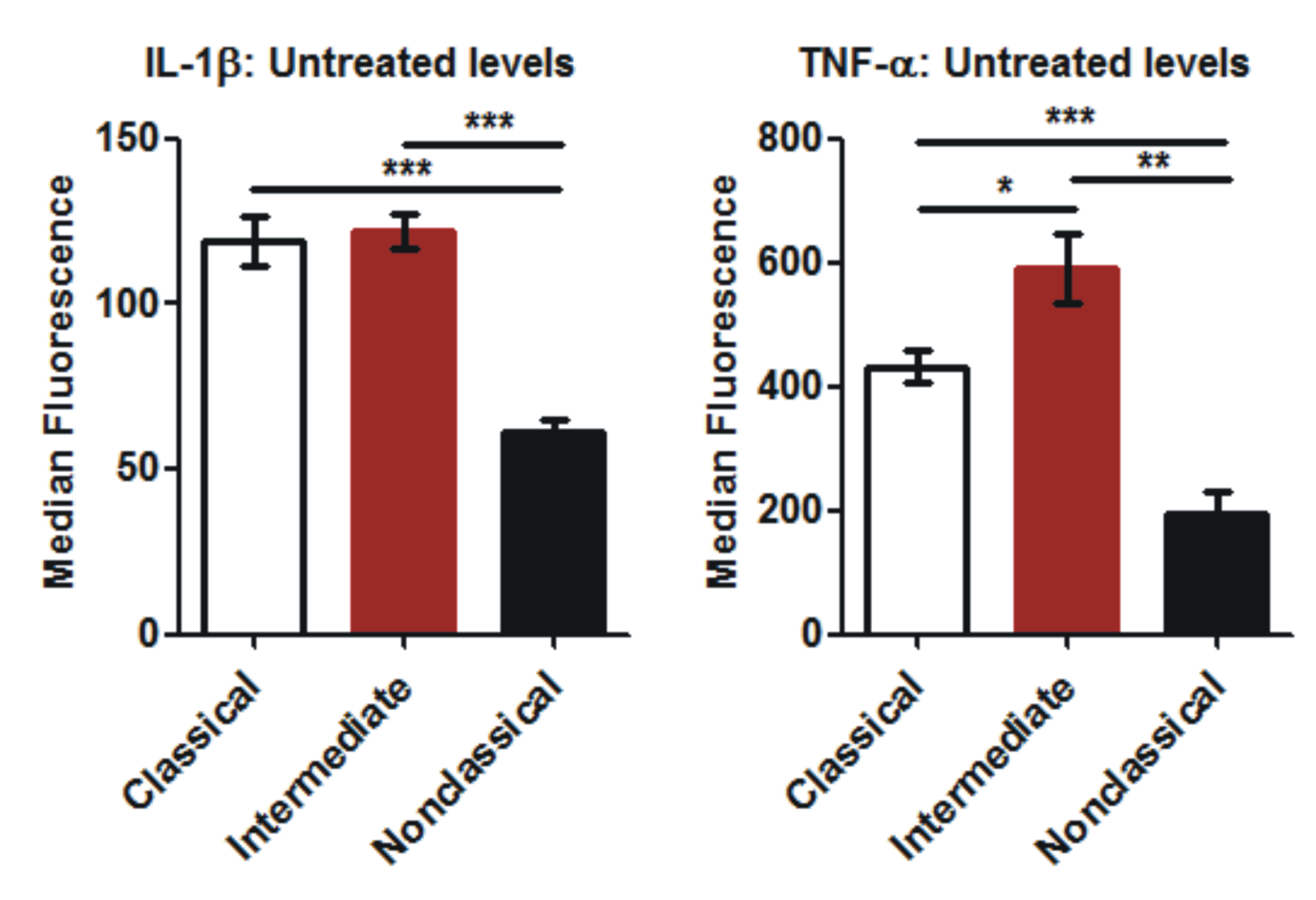


**Supplementary figure 2. Comparison of intracellular IL-1β and TNF-α between monocyte subsets at steady-state levels.** Whole blood obtained from healthy subjects (n=5) was left untreated for 1 hour along with Brefeldin A and then stained with a cocktail of fluorescently tagged antibodies to surface markers followed by fixation and permeabilization before staining with antibodies to intracellular cytokines. **P*<0.05, ***P*<0.01, ****P*<0.001 assessed by one-way ANOVA followed by Bonferroni’s post-hoc test.

**
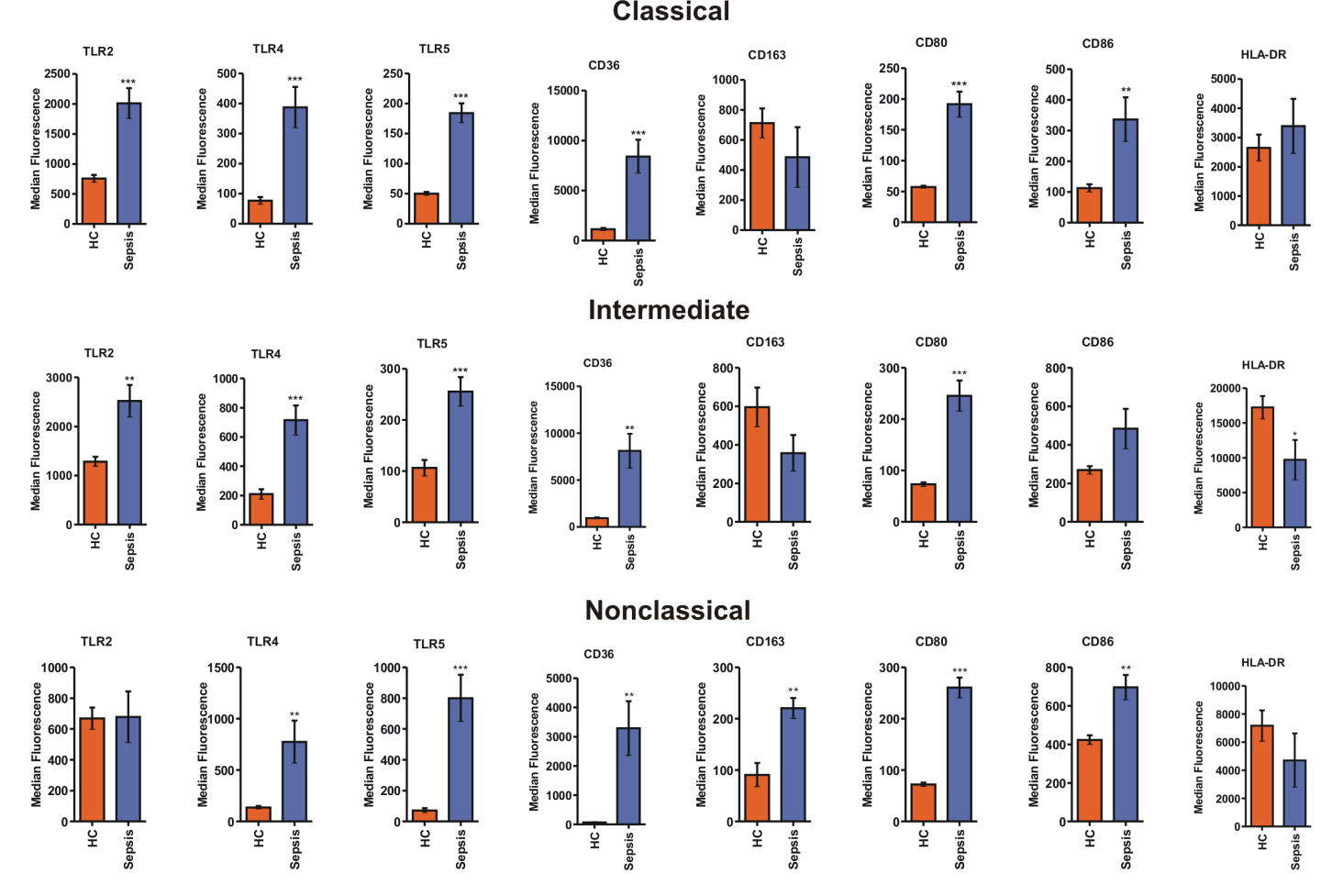
**

**Supplementary figure 3. Comparison of receptor expression on monocyte subsets between healthy individuals and Sepsis patients.** Whole blood obtained from either healthy subjects (n=7) or Sepsis patients (n=6) was stained with a cocktail of fluorescently tagged antibodies followed by RBC lysis and were analysed on a flow cytometer. **P*<0.05, ***P*<0.01, ****P*<0.001 assessed by unpaired t-test.

**
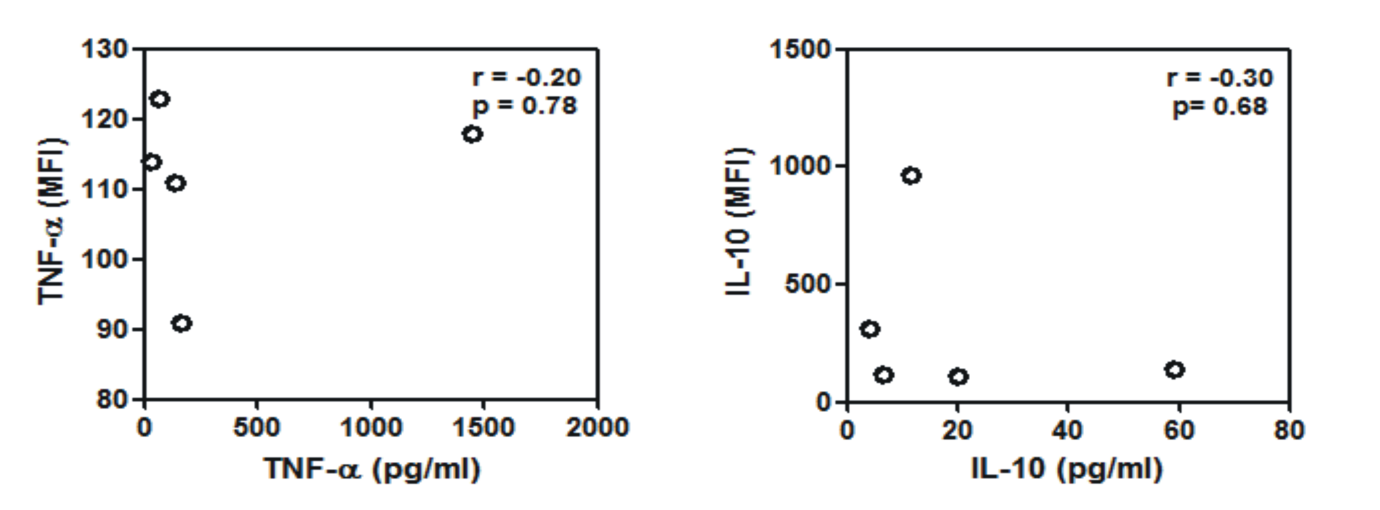
**

**Supplementary figure 4. Plasma and intracellular cytokines are not correlated in patients with sepsis.** Plasma was isolated from whole blood by centrifugation at 3000 rpm for 10 minutes. Plasma levels of TNF-α and IL-10 was measured by Bioplex suspension array system (Bio-rad) using manufacturer’s instructions. For measurement of intracellular cytokines, blood was first incubated with Brefeldin A at 1:1000 dilution for 1 hour. Post incubation, whole blood was stained with a cocktail of cell surface antibodies followed by fixation and permeabilization to stain for intracellular cytokines. Finally, the cells were washed and analysed on a flow cytometer. For TNF-α, MFI of only nonclassical subset and for IL-10, MFI of only intermediate subset is compared with total TNF-α and IL-10 in plasma. Data is representative of five individuals. Correlation was assessed using a nonparametric Spearman’s rank correlation test. MFI: Median Fluorescence Intensity.
